# Efficacy of transversus abdominis plane block on postoperative nausea and vomiting: a meta-analysis of randomized controlled trial

**DOI:** 10.1186/s12871-024-02469-x

**Published:** 2024-03-01

**Authors:** Jinfang Zeng, Aonan Hong, Zhen Gu, Jinjin Jian, Xiao Liang

**Affiliations:** 1 Department of Anesthesiology, Jiangnan University Medical Center, Affiliated Wuxi Clinical College of Nantong University, Wuxi, 214002 China; 2 Department of Anesthesiology, Affiliated Hospital of Nanjing, University of Chinese Medicine, Jiangsu Province Hospital of Chinese Medicine, Nanjing, 210000 China; 3 Department of Anesthesiology, Affiliated Hospital of Jiangnan University, Wuxi, 214002 China

**Keywords:** Transversus abdominis plane, Meta-analysis, Nausea, Vomiting

## Abstract

**Background:**

Postoperative nausea and vomiting (PONV) is a common postoperative complication, and Transversus abdominis plane (TAP) block can provide effective analgesia for surgical operation. However, but there is not enough evidence to prove its advantage for nausea and vomiting. The objective of this meta-analysis was to evaluate the efficacy of TAP block on PONV.

**Methods:**

Two independent researchers conducted searches for randomized controlled trials (RCTs) in PubMed, Embase, and Cochrane Central Register of Controlled Trials. We used Review Manager software for meta-analysis.

**Results:**

In this meta-analysis, twenty-six trials with 1981 patients were examined. The results showed that TAP block reduced postoperative nausea (Risk Difference (RD) = -0.10, 95% confidence interval (CI): -0.15 to -0.05) compared with no TAP block. TAP block reduced the dose of fentanyl (Standardized Mean Difference (SMD) = -1.17, 95% CI: -2.07 to -0.26) and morphine (SMD = -1.12, 95% CI: -2.10 to -0.13) compared with no TAP block, when the timing of administration was before surgery (RD = -0.13, 95% CI: -0.19 to -0.07). TAP block reduced postoperative nausea when the ropivacaine dosage is ≤ 100 mg (RD = -0.13, 95% CI: -0.21 to -0.06), bupivacaine dosage ≥ 100 mg ( RD = -0.08, 95% CI: -0.13 to -0.03), and when the ropivacaine concentration was ≤ 0.375% (RD = -0.11, 95% CI: -0.18 to -0.04). TAP block significantly reduced the incidence of nausea when the types of opioid drugs in PCA is tramadol (RD = -0.13, 95% CI: -0.24 to -0.03). TAP block could reduce the VAS (SMD= -0.99, 95% CI: -1.29 to -0.70) and reduce the time of extubation (SMD = -0.71, 95% CI: -1.34 to -0.08).

**Conclusion:**

The meta-analysis conducted in this study revealed that TAP block could reduce the incidence of PONV, and the efficacy of TAP block may be influenced by factors such as administration time, local anesthetic dosage and concentration, types of opioid drugs in PCA.

**Supplementary Information:**

The online version contains supplementary material available at 10.1186/s12871-024-02469-x.

## Introduction

As one of the most common complications after general anesthesia, postoperative nausea and vomiting (PONV) could increase morbidity and prolong hospital stay [1, 2]. Meanwhile, the increasing abdominal pressure during vomiting not only may increase the wound rupture rate, but vomiting may also cause electrolyte imbalance and acid-base disorder [3]. In addition, four clear risk factors that independently predicted PONV included smoking history, age and sex, motion sickness, and PONV history, which increased the risk by 20% respectively [4]. At the same time, the risk of PONV may also be related to anesthesia techniques, pre-anesthesia administration, and postoperative pain management [5, 6].

Transversus abdominis plane (TAP) block is performed either using a blind technique or ultrasonography [7]. In recent years, safe and accurate ultrasound-guided TAP block has been our most commonly used method [8]. TAP block is a widely used peripheral nerve block that blocks the body nerves supplying the anterior abdominal wall by deposing local anesthetics in the neurovascular plane between the internal oblique muscle and the transversus abdominis muscle layer [9, 10]. As effective constituents of multimode analgesia, TAP block are mainly used for postoperative analgesia in abdominal surgery. Some studies [11, 12] found that TAP block significantly decreased pain score and total opioid consumption. Similarly, Hosgood et al. [13] also claimed that TAP block reduced the early morphine requirement in a similar patient population, but some studies [14] found that TAP block did not decrease intra- and postoperative opioid consumption. The effects of TAP block on opioid sparing effects were both in the intraoperative as well as the postoperative period. Opioids, though very effective in perioperative pain management, may be associated with PONV, pruritus and respiratory depression. At present, some studies have demonstrated the efficacy of TAP block on reducing PONV, compared to no TAP block [15–17], however, others have not [18–20].

To the best of our knowledge, no quantitative analysis has been conducted on the effect of TAP block on PONV. As a result, we conducted a meta-analysis with the aim of exploring the efficacy of TAP block as an antiemetic agent.

## Methods

We conducted a meta-analysis to assess the effects of TAP block on PONV, as recommended by the PRISMA statement. The registration number of the study in PROSPERO is CRD42023420414. Ethical approval and patient consent are not required in a meta-analysis. Since very few patients vomit without experiencing nausea, and the incidence rates of PONV and postoperative nausea (PON) are similar, we consider PONV as a surrogate for PON if PONV is not reported in a trial. We evaluated nausea values in cases where PONV or PON occurred. The most commonly used time interval to measure the role of antiemetic is 24 h, when only longer or shorter time interval was reported, we utilized the interval closest to the 24-hour period. Nausea was assessed using a categorical scoring system (0 = none, 1 = mild, 2 = moderate, 3 = severe).

### Search approach and eligibility standards

The Cochrane library, Embase, and PubMed databases were systematically searched by Z.J.F. and L.X. for studies related to transversus abdominis plane or TAP, nausea, vomiting or PONV, and surgery, anesthesia or postoperative care. The search was conducted through March 22, 2023, and there were no language restrictions. In addition, the reference lists of original reports, review and case reports were checked to identify.

### Research selection

Data search included author name, publication year, anesthesia and surgery type/duration, interventions, cases of nausea/vomiting, and total patients. Two authors (G.Z. and J.J.J.) independently assessed articles for inclusion/exclusion criteria, with any disputes discussed by all authors.

### Inclusion criteria

Studies were included if they met all eligibility criteria, stated as: [1] population: adult patients (age ≥ 18 years) undergoing abdominal surgery under general anesthesia, [2] intervention: TAP block, If the control group was included in the article which compared TAP versus other type of nerve blocks, these articles would be included, [3] comparator: placebo or no intervention, [4] primary outcomes: incidence of nausea or vomiting; secondary outcomes: postoperative opioid consumption, the number of patients receiving rescue antiemetics, VAS, time of extubation and first flatus, satisfaction degree, duration of hospitalization, [5] study types: randomized controlled trials (RCTs).

### Exclusion criteria

[1] Registration number or abstract only; [2] Missing data; [3] Incorrect statistical analysis; [4] TAP block versus other nerve blocks.

### Information extraction and evaluation of bias risk

Two authors (H.A.N and G.Z) independently assessed study quality using the Cochrane Collaboration risk-of-bias tool. We evaluated six categories (selective reporting, incomplete result data and other biases, random sequence generation, allocation concealment, and blind methods). We classify each category as high risk, low risk, or unclear risk.

### Quality analysis of evidence

Quality of evidence was evaluated by GRADE (Grades of Recommendation, Assessment, Development, and Evaluation) system using the Guideline Development Tool.

### Outcome measures

Z-test was used to demonstrate whether the overall effect was significant. A p-value < 0.05 was considered statistically significant. A random-effect model was used. The combined risk difference (RD) was calculated to evaluate the efficacy of ropivacaine concentration, ropivacaine dosage, the types of opioid drugs in PCA, the dose of antiemetic, TAP block on nausea, vomiting, the time of administration, operation type, type of local anesthetic, bupivacaine concentration and dosage. The combined standardized mean difference (SMD) was used to evaluate the consumption of intraoperative opioids, the time of surgery and anesthesia, the time of extubation, the time of hospitalization, the time of first exhaust, VAS and satisfaction, with a confidence interval (CI) of 95%. Subgroup analyses were conducted based on the the type of surgery, type of local anesthetic, concentration of local anesthetic, the types of opioid drugs in PCA and administration time. The robustness of the results was analyzed through sensitivity analyses by only reanalyzing data from low risk and unclear risk studies.

## Results

### Study selection

**Fig. 1:**
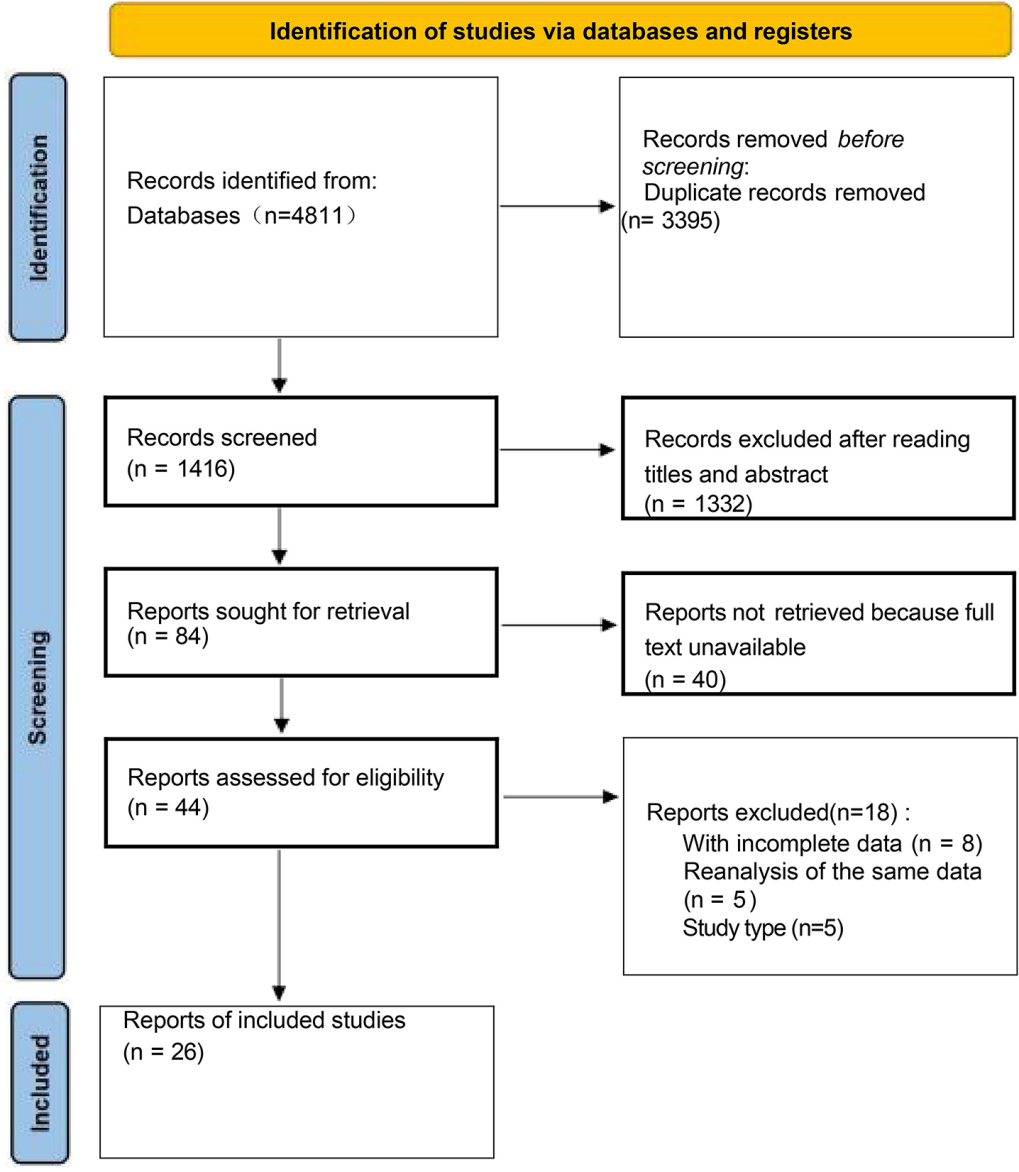
Flow diagram of the inclusion and exclusion process

As shown in the flow diagram (Fig. 1), the search of PubMed, Embase, Cochrane library, and reference lists yielded 4811 articles. Initially, 393 trials were discarded because they were not controlled trials by reading the titles. Then, 3002 trials were excluded for duplicates and 78 was review. Then, 499 trials did not satisfy the inclusion. Eighty-four papers were carefully read, and we found no related endpoints were reported in 58 papers, so they were excluded. Finally, 26 trials [15–40] that met the selection criteria were included in the meta-analysis.

### Study characteristic

Of all the included studies, 26 trials [15–40] explored the efficacy of TAP on PONV (Table 1). All the included documents are from 2011 and later. The number of cases of local anesthesia with bupivacaine was 890 cases and ropivacaine was 814 cases. There were laparoscopic surgery treatments of 1025 cases and non-laparoscopic surgery treatments of 751 cases. Moreover, the timing of administration was mostly before surgery.

**Table 1:** General information of patients with incidence of postoperative nausea and vomiting

Author	Year	Age	Sex (Male/Female)	Type of sugery	Comparisons	Timing of administration	Nausea	Vomiting	Total
Aniskevich, S	2014	23–79 years	6/4	Laparoscopic hand-assisted nephrectomy	TAP 0.5% ropivacaine 100 mg	Before surgery	5	2	10
			6/5		Control saline 20 ml		10	2	11
Bharti, N	2011	18–60 years	14/6	Colorectal surgery	TAP 0.25% bupivacaine 50 mg	At the end of surgery	8	4	40
			14/6		Control saline 20 ml		4	-	40
Bhattacharjee, S	2014	-	0/45	Total abdominal hysterectomy by a lower abdominal transverse incision	TAP 0.25%bupivacaine 100 mg	Before anesthesia	8	-	45
		-	0/45		Control saline 40 ml		12	-	45
Cevikkalp, E	2023	18–70 years	11/24	Laparoscopic cholecystectomy	TAP 0.25%bupivacaine 100 mg	Before anesthesia	9	-	35
			7/27		Control saline 40 ml		9	-	34
Covotta, M	2020	≥ 18 years	30/18	Robotic partial nephrectomy	TAP 0.5% ropivacaine 150 mg	After induction of general anesthesia	9	-	48
			22/26		Control nothing		18	-	48
Geng, Z. Y	2023	18–65 years	0/32	Elective open gynecological surgery	TAP 0.375% ropivacaine 75 mg	After induction of anesthesia and before surgery	8	7	32
			0/32		Control saline 20 ml		16	7	32
Guo, J. G	2018	18–65 years	25/10	Open liver resection	TAP0.375% ropivacaine 75 mg	Before anesthesia	2	2	35
			23/12		Control saline 20 ml		8	4	35
Hutchins, J	2014	-	0/30	Robotic assisted hysterectomy	TAP 0.25%bupivacaine 37.5 mg	After induction of general anesthesia	6	-	30
		-	0/30		Control nothing		16	-	30
Kawahara, R	2015	≥ 18 years	0/60	Gynecologic laparoscopic surgery	TAP 0.375% ropivacaine 75 mg	Following general anesthesia	11	-	60
			0/59		Control saline 20 ml		21	-	59
Keller, D	2014	> 18 years	18/23	Laparoscopic colorectal Surgery	TAP0.5% bupivacaine 150 mg	At the completion of the procedure	10	-	41
			16/22		Control saline 30 ml		9	-	38
Kim, M. G	2014	≥ 18 years	31/2	Laparoscopic totally extraperitoneal hernia repair	TAP 0.375% ropivacaine112.5 mg	After induction of general anesthesia	6	-	33
			33/4		Control nothing		5	-	37
Korkmaz Toker, M	2019	18–65 years	0/30	Laparoscopic hysterectomy for benign gynecologic pathologies	TAP 0.375% bupivacaine 150 mg	Before the initiation of surgery	11	-	30
			0/30		Control saline 40 ml		15	-	30
Li, X	2019	18–70 years	32/20	Retroperitoneoscopic renal surgery	TAP0.4% ropivacaine 120 mg	After induction of general anesthesia	17	-	52
			31/20		Control saline 30 ml		15	-	51
Lochel, J	2021	≥ 18 years	-	Periacetabular osteotomy	TAP 0.75% ropivacaine 150 mg	After induction of general anesthesia	9	1	21
			-		Control nothing		9	1	20
Lu, X	2020	> 18 years	59/4	Laparoscopic hepatectomy	TAP 0.25% ropivacaine 100 mg	At the end of surgery	21	15	63
			45/18		Control nothing		22	16	63
Ma, J	2018	18–75 years	18/11	Laparoscopic colectomy	TAP 0.375% ropivacaine 75 mg	After induction of general anesthesia	4	-	29
			17/11		Control saline 20 ml		6	-	28
McKeen, D. M	2014	≥ 18 years	0/35	Cesarean delivery	TAP 0.25% ropivacaine 100 mg	At the end of surgery	-	2	35
			0/39		Control saline 40 ml		-	2	39
Petersen, P. L	2012	18–75 years	9/28	Laparoscopic cholecystectomy	TAP 0.5% ropivacaine 100 mg	Before surgical incision	-	8	37
			12/25		Control saline 20 ml		-	13	37
Reisener, M. J	2021	≥ 18 years	54/75	Anterior or lateral lumbar fusion	TAP 0.5% bupivacaine 100 mg	After induction of general anesthesia	4	-	129
			61/60		Control nothing		15	-	121
Sivapurapu, V	2021	18–80 years	28/2	Laparoscopic total extraperitoneal repair of unilateral hernia surgeries	TAP 0.25% levobupivacaine 45 mg	After induction of anesthesia	7	-	30
			26/4		Control nothing		21	-	30
Skjelsager, A	2013	18–80 years	23/0	Open radical prostatectomy	TAP 0.75% ropivacaine 30 mg	At the end of the surgery	-	7	23
			24/0		Control saline 40 ml		-	8	24
Soltani Mohammadi, S	2014	15–65 years	12/10	Kidney recipients	TAP0.25% bupivacaine 37.5 mg	After induction of anesthesia	0	-	22
			14/8		Control saline 15 ml		2	-	22
Tan, T	2012	> 18 years	0/20	Caesarean delivery	TAP 0.25% levobupivacaine 50 mg	After the procedure, before the patients awakened	2	0	20
			0/20		Control nothing		4	1	20
Tupper-Carey, D. A	2017	> 21 years	21/8	Urgent laparoscopic appendicectomy	TAP 0.5%bupivacaine 50 mg	After the procedure, before the patients awakened	11	-	29
			25/4		Control saline 10 ml		7	-	29
Zhang, J	2020	-	12/12	Laparoscopic hepatectomy	TAP 0.3% ropivacaine 180 mg	At the end of surgery	7	-	24
		-	13/19		Control saline 60 ml		11	-	23
Zhang, L	2023	20–60 years	0/35	Elective gynecological laparotomy	TAP0.375% ropivacaine 150 mg	After the induction of anesthesia	6	2	35
			0/37		Control saline 40 ml		12	5	37

### The methodological quality of the included studies

**Fig. 2:**
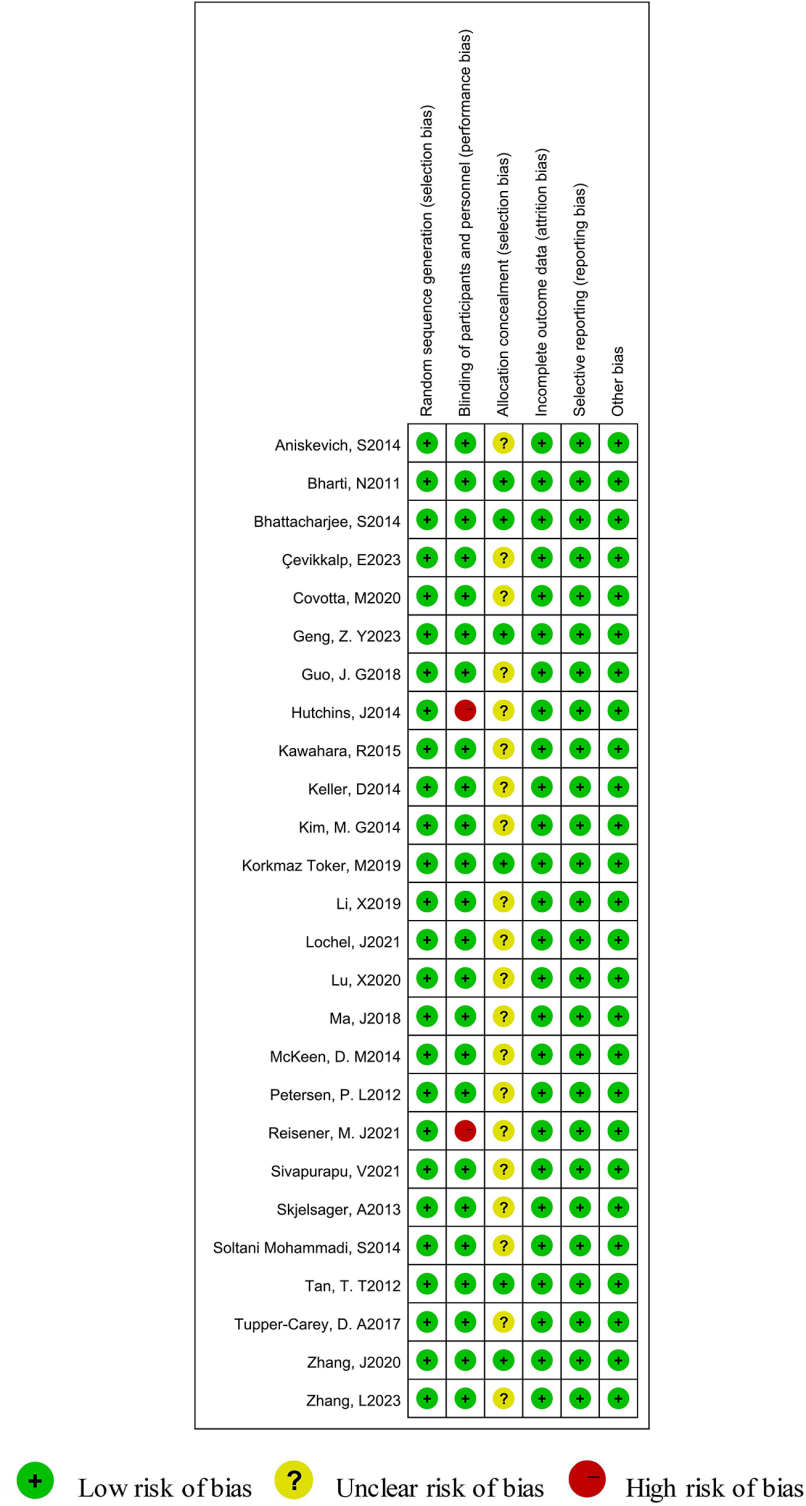
Summary of the risk of bias of the included studies

A low risk of overall risk of bias for included 26 trials [15–40]. Twelve studies [29–40] employed random number tables, six study (16, 18–20, 22–23) used sealed envelopes, and eight studies [15, 17, 21, 24–28] adopted computer generated random numbers. Two study [17, 32] did not mention the method used to blind the subjects. We judged the study to be “high risk of bias”’. Only 6 studies [16, 18, 22, 26, 36, 40] described the allocation concealment. Most of the studies reviewed lacked sufficient details in allocation concealment, in such cases, we were conservative in our risk of bias evaluation by tending to classify trials as having an “unclear risk of bias”. In addition, all studies [15–40] reported the completion of the trial without withdrawals, and all the studies [15–40] reported all the end points mentioned in the Methods section (reporting bias). Other bias might exist in all trials [15–40]. An overview of the risk of bias was summarized in Fig. 2.

### Quality of evidence

GRADE system grades of evidence showed that having the serious risk of bias in some of those studies, and with that CIs showed minimal or no overlap, and the publication bias was not assessed because of the limit of the amount of included studies, all studies were designed with randomized mothed, quality of efficacy of TAP block on PONV was evaluated as the very low-certainty evidence (Supplementary Table 1).

### Results of meta-analysis

**Fig. 3:**
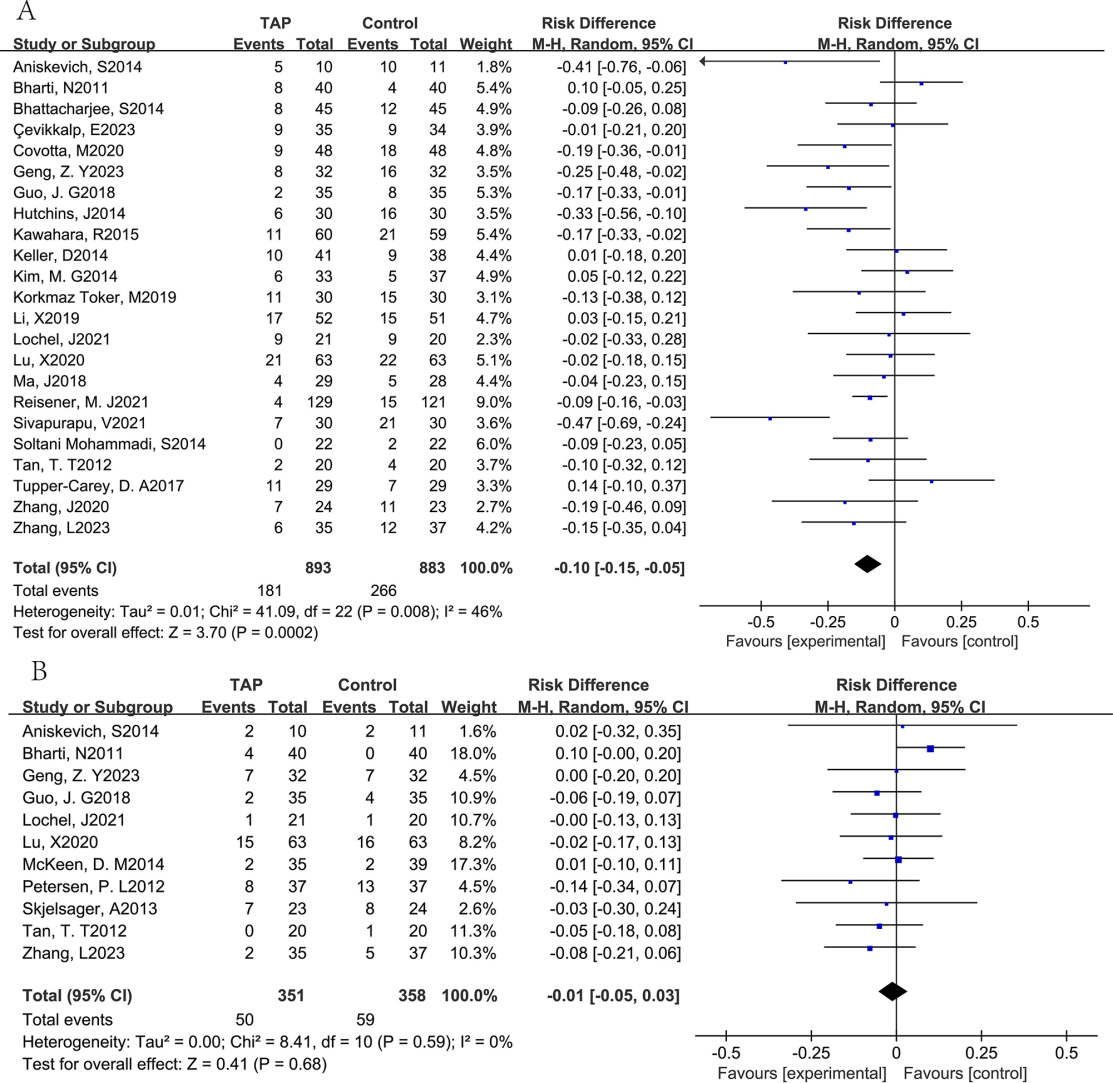
Results of the incidence of postoperative nausea (**A**) and vomiting (**B**)

**Fig. 4:**
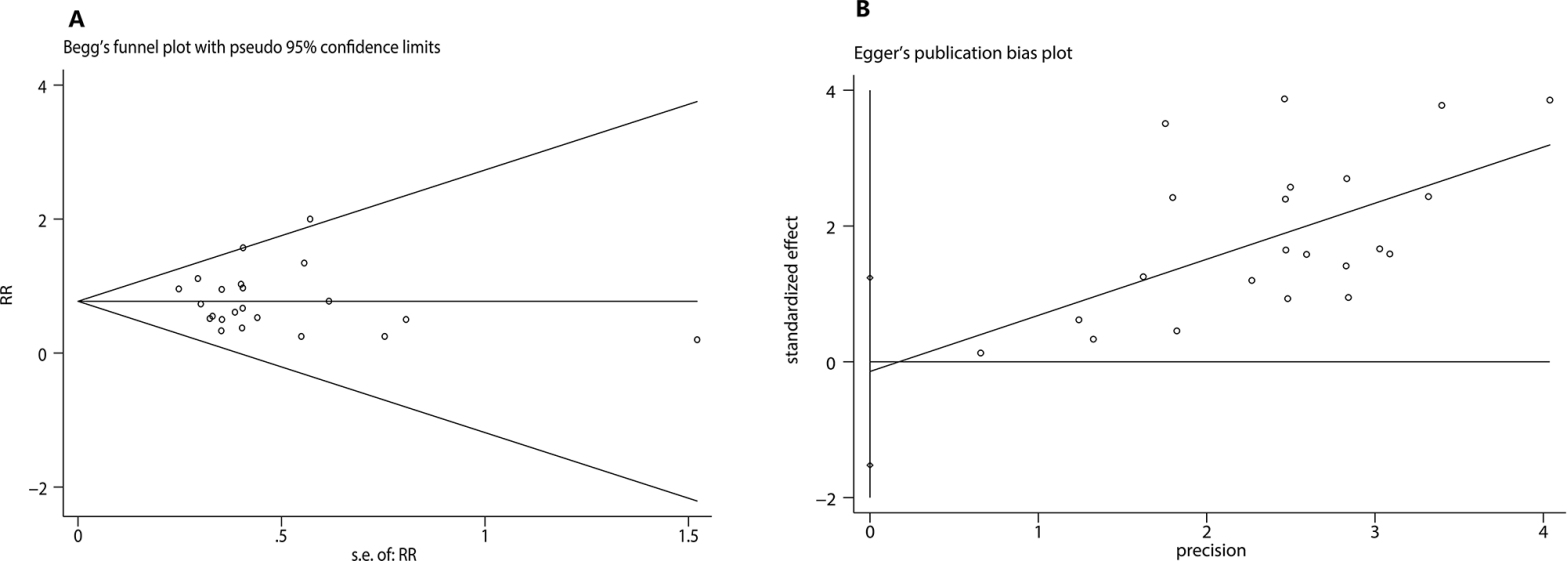
Results of the Begg’s test and Egger’s test

*TAP block on PONV*: Twenty-three trials [15–30, 32, 23, 35–38, 40], including 1,776 patients, investigated the efficacy of preventing nausea, meanwhile vomiting was detected in eleven trials [15, 18, 22, 23, 28, 29, 31, 34, 37, 39, 40] including 709 patients, by comparing TAP block with no TAP block. The incidence of nausea (pooled RD = -0.10, 95% CI: -0.15 to -0.05) in the TAP group was significantly lower than the control group, and the incidence of vomiting was not significantly lower than the control group. (pooled RD = -0.01, 95% CI: -0.05 to 0.03) (Fig. 3). And Begg’s test with *P* = 0.771 and Egger’s test with *P* = 0.832 suggested that no significant publication bias existed in the comparisons of nausea between TAP block with no TAP block (Fig. 4). Further, factors that affected nausea and vomiting were evaluated through subgroup analysis below:

*Time of administration*: TAP block significantly reduced the incidence of nausea (pooled RD of 17 trials [15–17, 19, 22–24, 26–28, 30, 32, 33, 35, 37, 38]: -0.13, 95% CI: -0.19 to -0.07) when the timing of administration was before surgery, but not after surgery (pooled RD of 6 trials [18, 20, 25, 29, 36, 40]: 0.01, 95% CI: -0.07 to 0.09) (Supplementary Fig. 1A).

*Operation type*: TAP block significantly reduced the incidence of nausea (pooled RD of nine trials [16, 18, 22, 23, 28, 32, 35, 37, 40]: -0.09, 95% CI: -0.14 to -0.04) in non-laparoscopic surgery, but also in laparoscopic surgery (pooled RD of 14 trials [15, 17, 19–21, 24–27, 29, 30, 36, 41]: -0.11, 95% CI: -0.19 to -0.02) (Supplementary Fig. 1B).

*Type of local anesthetic*: TAP block significantly reduced the incidence of nausea (pooled RD of 11 trials [15, 19, 21–24, 27–30, 36]: -0.01, 95% CI: -0.18 to -0.03) when the local anesthetic was ropivacaine, and bupivacaine (pooled RD of 11 trials [16–18, 20, 26, 32, 33, 35, 38, 40]: -0.09, 95% CI: -0.17 to -0.00) (Supplementary Fig. 2).

*Local anesthetic dosage*: TAP block significantly reduced the incidence of nausea when the ropivacaine dosage was ≤ 100 mg(pooled RD of seven trials [15, 22–24, 29, 30, 40]: -0.13, 95% CI: -0.21 to -0.06) and bupivacaine dosage ≥ 100 mg (pooled RD of five trials (16, 25–26, 29, 32, 38): -0.08, 95% CI: -0.13 to -0.03) ,but not when the ropivacaine dosage was > 100 mg (pooled RD of six trials [19, 21, 27, 28, 36, 37] : -0.07, 95% CI: -0.16 to 0.02) and bupivacaine dosage <100 mg (pooled RD of four trials [17–21, 20, 35] : -0.04, 95% CI: -0.23 to 0.14) (Supplementary Fig. 3A,C).

*Local anesthetic concentration*: TAP block significantly reduced the incidence of nausea when the ropivacaine concentration was ≤ 0.375%(pooled RD of eight trials [19, 22–24, 29, 30, 36, 37]: -0.11, 95% CI: -0.18 to -0.04), but not > 0.375% (pooled RD of four trials [15, 21, 27, 28]: -0.12, 95% CI: -0.29 to 0.05) (Supplementary Fig. 3B).

*Types of opioid drugs in PCA*: TAP block significantly reduced the incidence of nausea when the types of opioid drugs in PCA was tramadol (pooled RD of threet trials [16, 24, 26]: -0.13, 95% CI: -0.24 to -0.03) (Supplementary Fig. 4).

*Cases with antiemetic or satisfaction degree*: Application of TAP block did not reduce the dose of antiemetic (pooled RD of seven trials [15, 18, 22, 24, 27, 39, 40]: -0.07, 95% CI: -0.16 to 0.01) compared with no TAP block, and could not increase the satisfaction degree (pooled SMD of two trials [37, 38]:0.33, 95% CI: -0.01 to 0.66). (Supplementary Fig. 5).

**Fig. 5:**
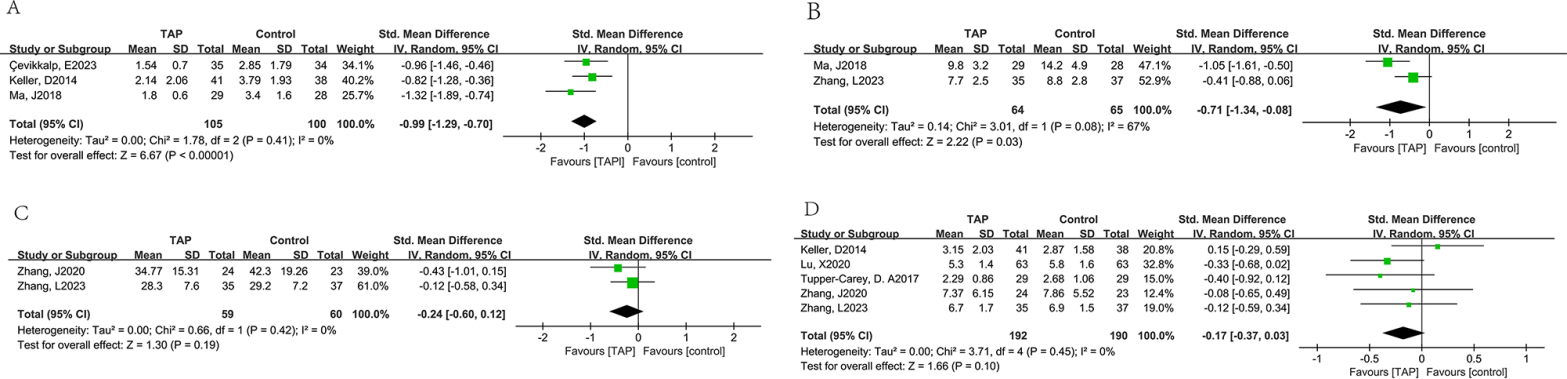
Results VAS (**A**), the time of extubation (**B**), first flatus (**C**) and the duration in the hospital(D)

*VAS, time of extubation, first flatus and duration of hospitalization*: TAP block could reduce the VAS (pooled SMD of three trials [25, 30, 38]: -0.99, 95% CI: -1.29 to -0.70) and reduce the time of extubation (pooled SMD of two trials [30, 37]: -0.71, 95% CI: -1.34 to -0.08) (Fig. 5A,B), although TAP block could not reduce the time of first flatus (pooled SMD of two trials [36, 37]: -0.24, 95% CI: -0.60 to 0.12) (Fig. 5C), and could not reduce the duration of hospitalization (pooled SMD of five trials [19, 20, 29, 36, 37]: -0.17, 95% CI: -0.37 to 0.03)(Fig. 5D).

**Fig. 6:**
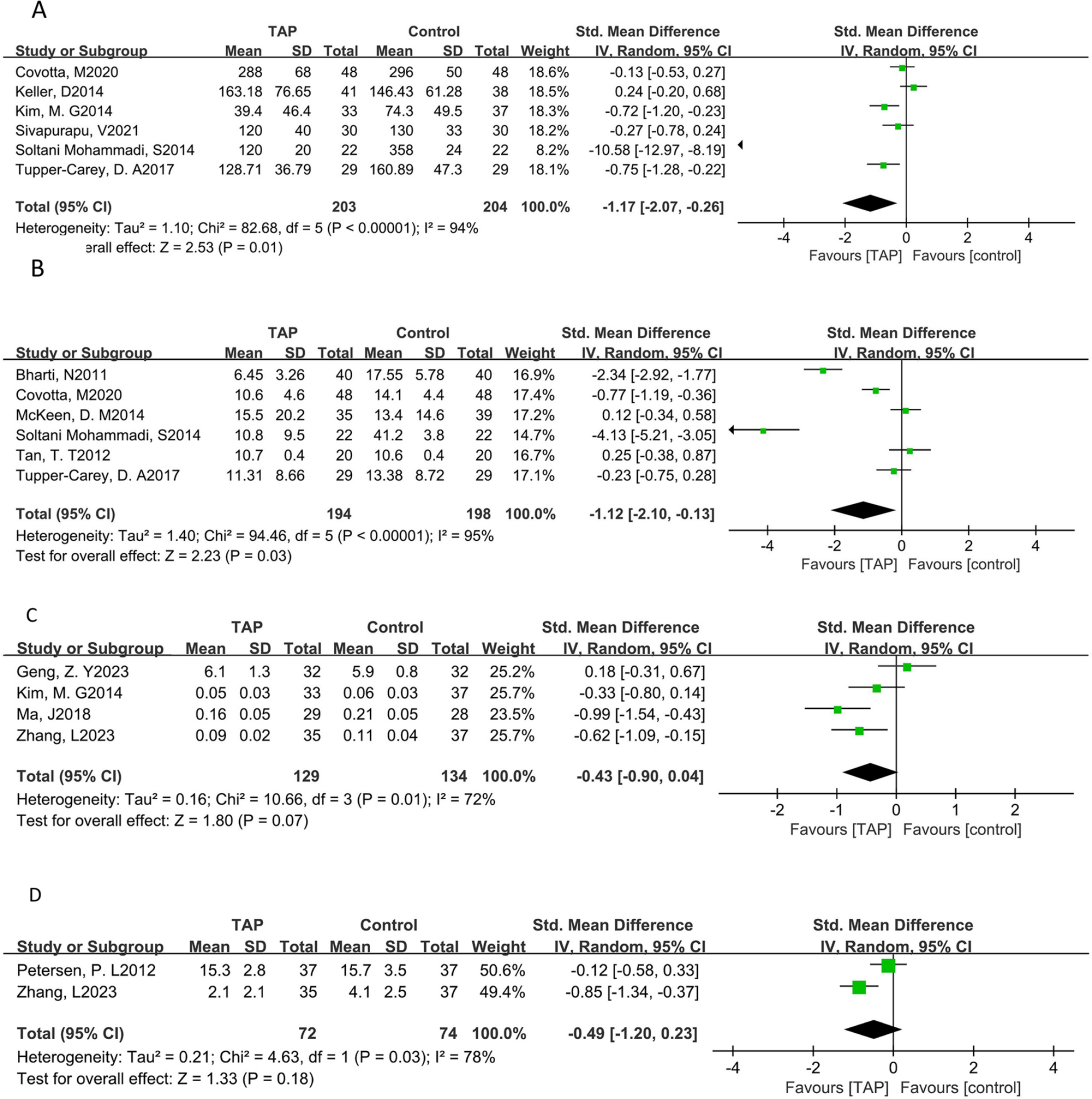
Results of the consumption of fentanyl (**A**), morphine (**B**), remifentanil (**C**) and sufentanil (**D**)

*Consumption of fentanyl, morphine, remifentanil and sufentanil*: TAP block could reduce the consumption of fentanyl (pooled SMD of six trials [19–21, 25, 33, 35]: -1.17, 95% CI: -2.07 to -0.26) (Fig. 6A) and morphine (pooled SMD of six trials [18, 20, 21, 31, 35, 40]: -1.12, 95% CI: -2.10 to -0.13) (Fig. 6B), although TAP block could not reduce the consumption of remifentanil and sufentanil (pooled SMD of four trials [19, 22, 30, 36]: -0.43, 95% CI: -0.90 to 0.04 and pooled SMD of two trials [37, 39]: -0.49, 95% CI: -1.20 to 0.23), but there was a trend. (Fig. 6C, D).

## Discussion

PONV is not a new issue in anesthesia, but a long-standing problem that has a significant impact on patients, delays discharge, increases hospital costs, and increases patients’ economic burden [41–43]. PONV could even destroy the balance of water and electrolyte, and in severe cases, it could lead to asphyxia and pneumonia. Although extensive research had been conducted, PONV remains a challenge for healthcare professionals due to its complex mechanisms. In clinical practice, ondansetron combined with dexamethasone was often used as a basic antiemetic drug for preventive antiemetic, but drug antiemetic could not completely relieve PONV, and drug therapy has side effects and contraindications. Therefore, it was particularly important to pay attention to non-drug treatment of PONV. Aim of our current meta-analysis was to evaluate the efficacy of TAP blockers in preventing PONV.

The main findings were as follows: (1) The incidence of PONV was lower in patients receiving TAP block without high risk factors. (2) TAP block administrated before surgery reduced the incidence of nausea in non-laparoscopic and laparoscopic surgery, but not after surgery. (3) TAP block reduced the incidence of nausea with the dosage ≤ 100 mg and concentration ≤ 0.375% of ropivacaine and bupivacaine dosage ≥ 100 mg. (4) TAP block significantly reduced the incidence of nausea when the type of opioid drugs in PCA is tramadol. (5) TAP block did not reduce the dose of antiemetic compared with no TAP block, and could not increase the satisfaction degree. (6) TAP block could reduce the VAS and reduce the time of extubation, but could not reduce the time of first flatus, and could not reduce the duration of hospitalization. (7) TAP block could reduce the consumption of fentanyl and morphine.

In the past few decades, most acute pain related to surgery has been treated with opioid drugs for pain relief [44]. Although they are very effective in perioperative pain management opioids may have relation with PONV, delirium, sedation, constipation, tolerance, respiratory depression [45]. Recently, various regional blocks had been applied in surgery to reduce opioid consumption and achieve desired pain control [46, 47]. Many clinical trials had confirmed the effectiveness of TAP in pain control as part of multimodal postoperative analgesia, but it was a relatively new regional block, although its mechanism was still controversial [48].

The injection of local anesthetics into the TAP blocks sensory nerve afferents to the skin, muscle, and parietal peritoneum of the anterior abdominal wall innervated by T_7 − 12_ and L_1_. TAP block may provide more effective pain relief and minimize postoperative opioid consumption, thus preventing opioid-related complications, promote recovery of bowel function and decrease PONV [49]. When comparing results among different trials, it is crucial to take the surgical technique, the block approach and the time of block into consideration. The onset of the sensory block appeared to be relatively slow which might take up to 60 min to reach maximal effect, so ideally the block was placed before the start of surgery with adequate time for the onset of analgesia [37]. So when the TAP block was placed before surgery, TAP block minimized opioid consumption. Accordingly, we found that TAP block significantly reduced the incidence of nausea when the timing of administration was before surgery, but not after surgery. In our study, we found that if we used a higher concentration (> 0.375%) of ropivacaine, the efficacy of TAP block on PONV would be inferior consistent with the findings of the previous studies [50–52] which indicated that the postoperative analgesic effect would be compromised if a higher concentration of local anesthetic was used. As we know, tramadol had a higher incidence of nausea and vomiting than morphine, and we found that TAP block significantly reduced the incidence of nausea when the type of opioid drugs in PCA is tramadol.

The risk factors of PONV could be divided into three categories, including patient factors, anesthesia techniques, and surgical related risk factors. Risk factors related to anesthesia techniques include the use of inhalers within 0 to 2 h, and the use of opioids during and after surgery. Surgical risk factors include long-term surgery and different types of surgery [53–55], and it had been confirmed that TAP block could reduce the duration of surgery which might be one of the reasons to reduce the incidence of PONV. In the fourth consensus guideline for the management of PONV, opioids were recognized as a risk factor for PONV which showed dose dependency. High-level evidence recommends reducing the using dose of opioid and combining multimodal analgesia to prevent PONV [56]. Indeed, we demonstrated that the application of TAP block did reduce the consumption of opioids, while the reduced VAS scores is consistent with the results of Zhang et al. and Bacal et al. [57, 58]. Noteworthy, the incidence of PONV was significantly lower in our TAP group, as compared to other studies, which could be explained by the lower 24-hour analgesic usage postoperatively.

The mechanistic reasons for the reduction of PONV by TAP might be as follows. First,, TAP could effectively relieve pain. We know that surgical trauma can cause postoperative pain in patients, and pain can lead to PONV in patients with mental tension. Poor postoperative pain control could not only lead to unpleasant subjective feelings of patients, but also cause PONV, hyperalgesia, respiratory dysfunction and other complications. Therefore, TAP could reduce PONV by reducing postoperative pain. Second, TAP could reduce opioid consumption. Opioids, such as morphine and fentanyl, play an analgesic effect by stimulating opioid receptors in the spinal cord, medulla oblongata and thalamus, and also activate opioid receptors in the medulla oblongata vomiting center, which leads to PONV, therefore, TAP could reduce opioids and its side effect PONV. Third, surgical operations could produce tissue trauma and inflammation. Increasing the duration of surgery appears to be the one consistent independent risk factor for PONV [59] .We found that TAP could reduce the duration of surgery, than reduce tissue trauma, and reduced nausea and vomiting. Last, Firoozabadi et al. [60] found that reduce mental relaxation can be used as an adjunct to deal with PONV .Therefore, we hypothesized that TAP could relieve pain, relax patients’ mind and reduce PONV.

### Limitations and suggestion for practice

This meta-analysis had several limitations. First, according to the GRADE system, the certainty of our findings ranked very low across different outcomes, the main limiting factors that contribute to the low quality included the serious risk of bias. Second, the total number of trials included was relatively large, but the number of subgroups, such as surgical type, drug concentration, etc., were still small, making it impossible to ensure conclusive results. Third, the high risk factors of PONV, such as the past history of motion sickness and non-smokers, were difficult to find in the whole literature, so we failed to take them as the third evaluation item.

## Conclusion and recommendations

In summary, TAP block decreases opioid consumption, prevents hemodynamic responses to surgical stimuli and also provided effective postoperative analgesia, improved pain scores, reduced the incidence of PONV, extubation and hospital times, meanwhile, improved satisfaction degree. These advantages may be of great importance undergoing surgical procedures to assure safe and rapid postoperative recovery. In the light of all these findings, TAP block could be considered as a safe and proper manner with few adverse effects.

### Electronic supplementary material

Below is the link to the electronic supplementary material.

Supplementary Material 1

## Data Availability

The datasets supporting the conclusions of this article are included within the Article.

## Abbreviations

ASA: American Society of Anesthesiologists classification
TAP: transversus abdominis plane
PACU: Post-anesthetic care unit
PCA: Patient-controlled analgesia
PONV: Postoperative nausea and vomiting
RCTs: randomized controlled trials
