# Supplementary material for: Efficacy of transversus abdominis plane block on postoperative nausea and vomiting: a meta-analysis of randomized controlled trial
Source: BMC Anesthesiol. 2024 Mar 1;24:87. doi: 10.1186/s12871-024-02469-x (PMC10905943; doi:10.1186/s12871-024-02469-x)
Supplement: Supplementary file 1 — Supplementary Material 1 [file 12871_2024_2469_MOESM1_ESM.docx]

**Supplementary figure 1.** Results of subgroup analysis of the incidence of postoperative nausea by time of administration (A) and operation type (B).

**
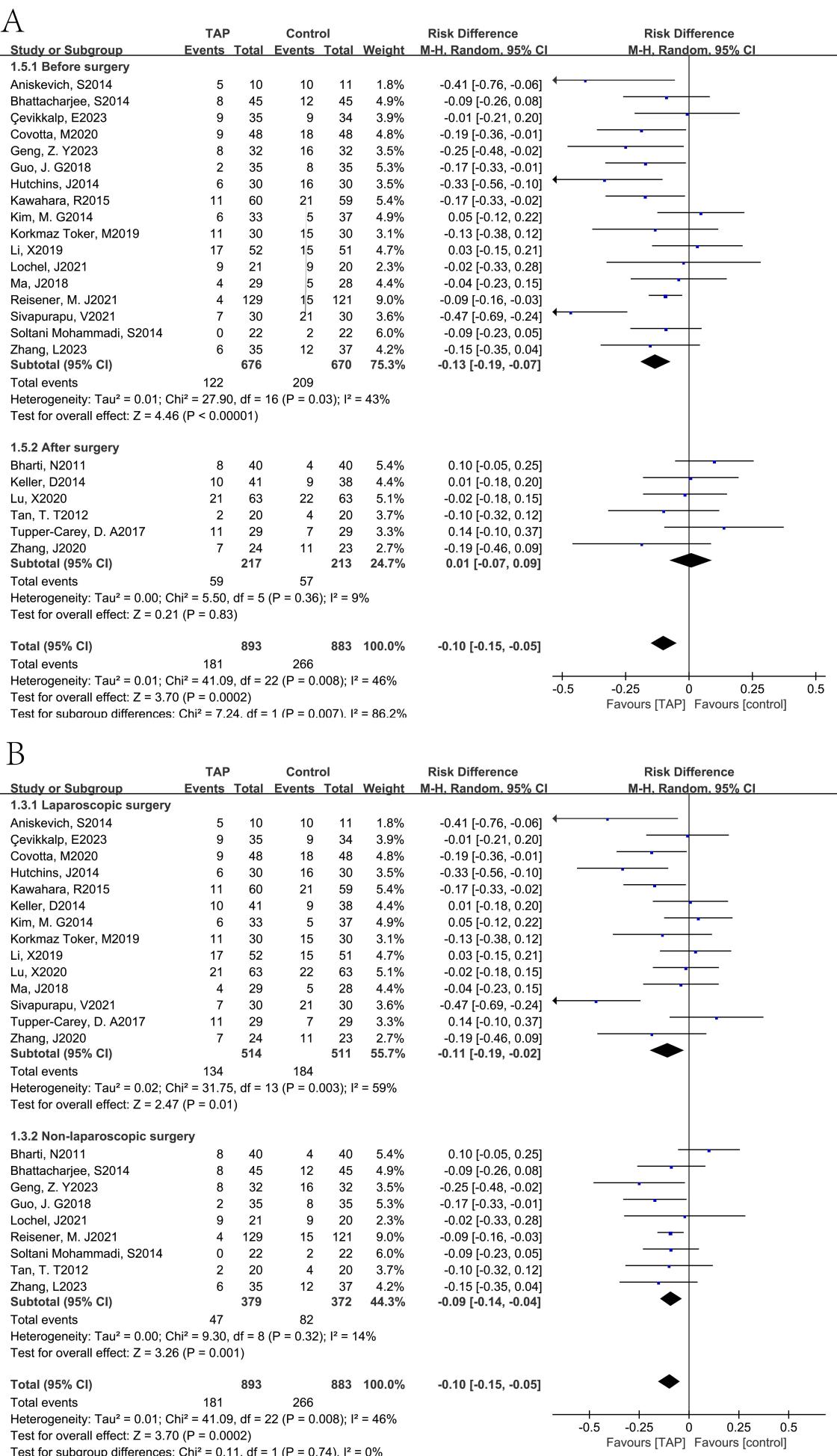
**

**Supplementary figure 2.** Results of subgroup analysis of the incidence of postoperative nausea by type of local anesthetic .


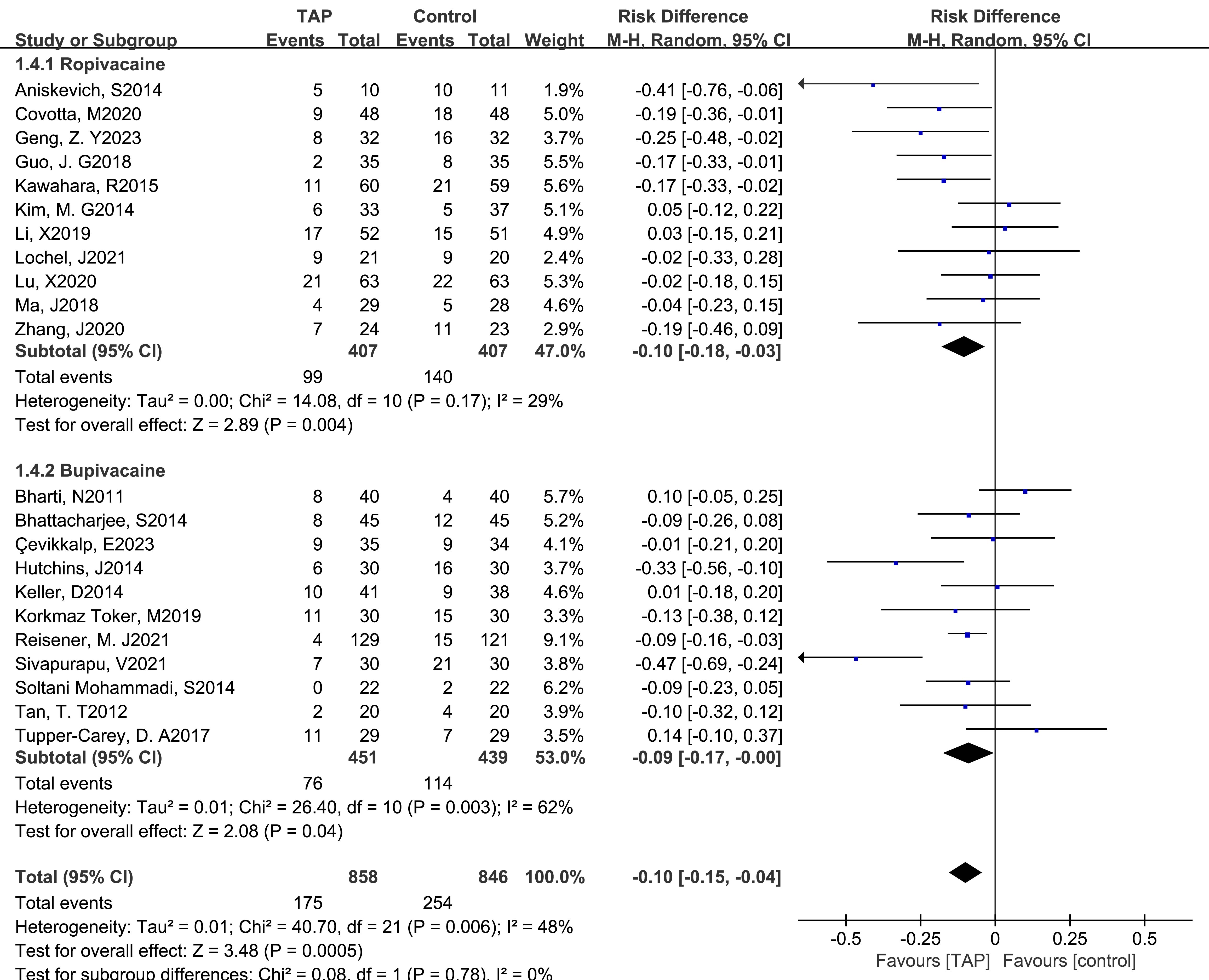


**Supplementary figure 3.** Results of subgroup analysis of the incidence of postoperative nausea by local anesthetic dosage(A,C) and local anesthetic concentration(B,D).


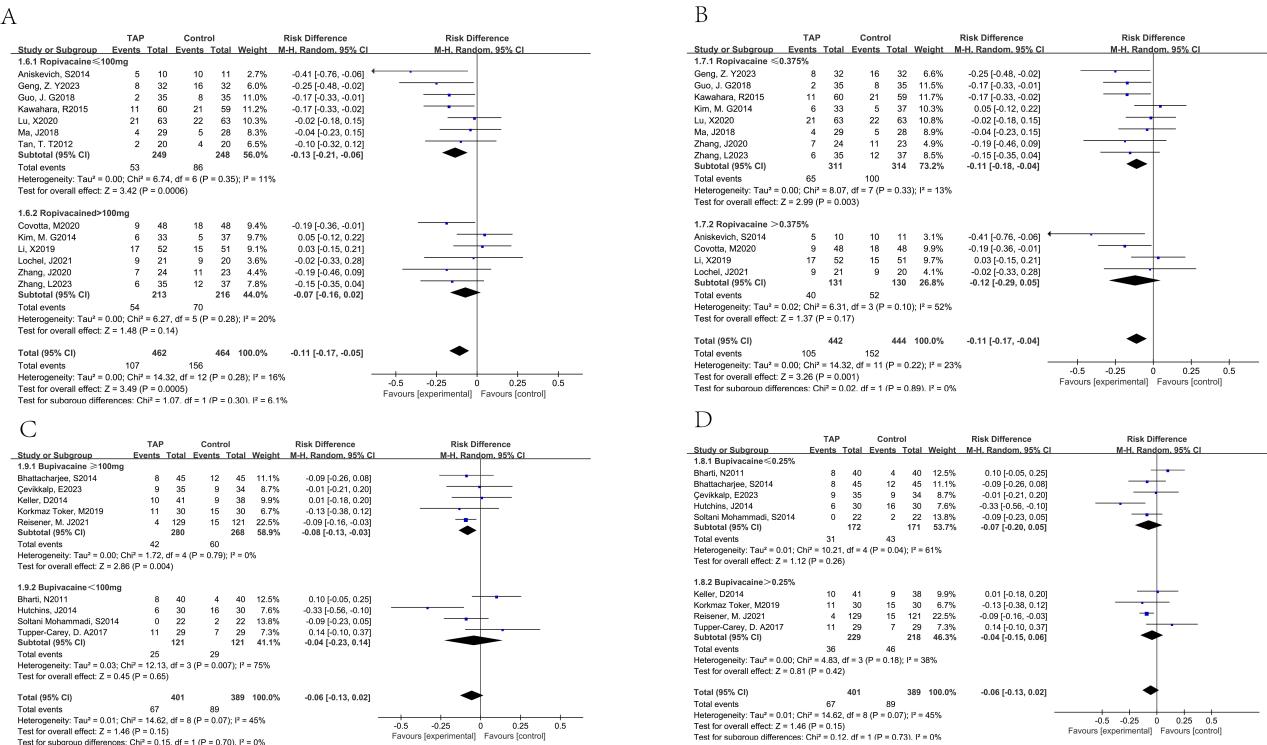


**Supplementary figure 4.** Results of subgroup analysis of the incidence of postoperative nausea by types of opioid drugs in PCA.


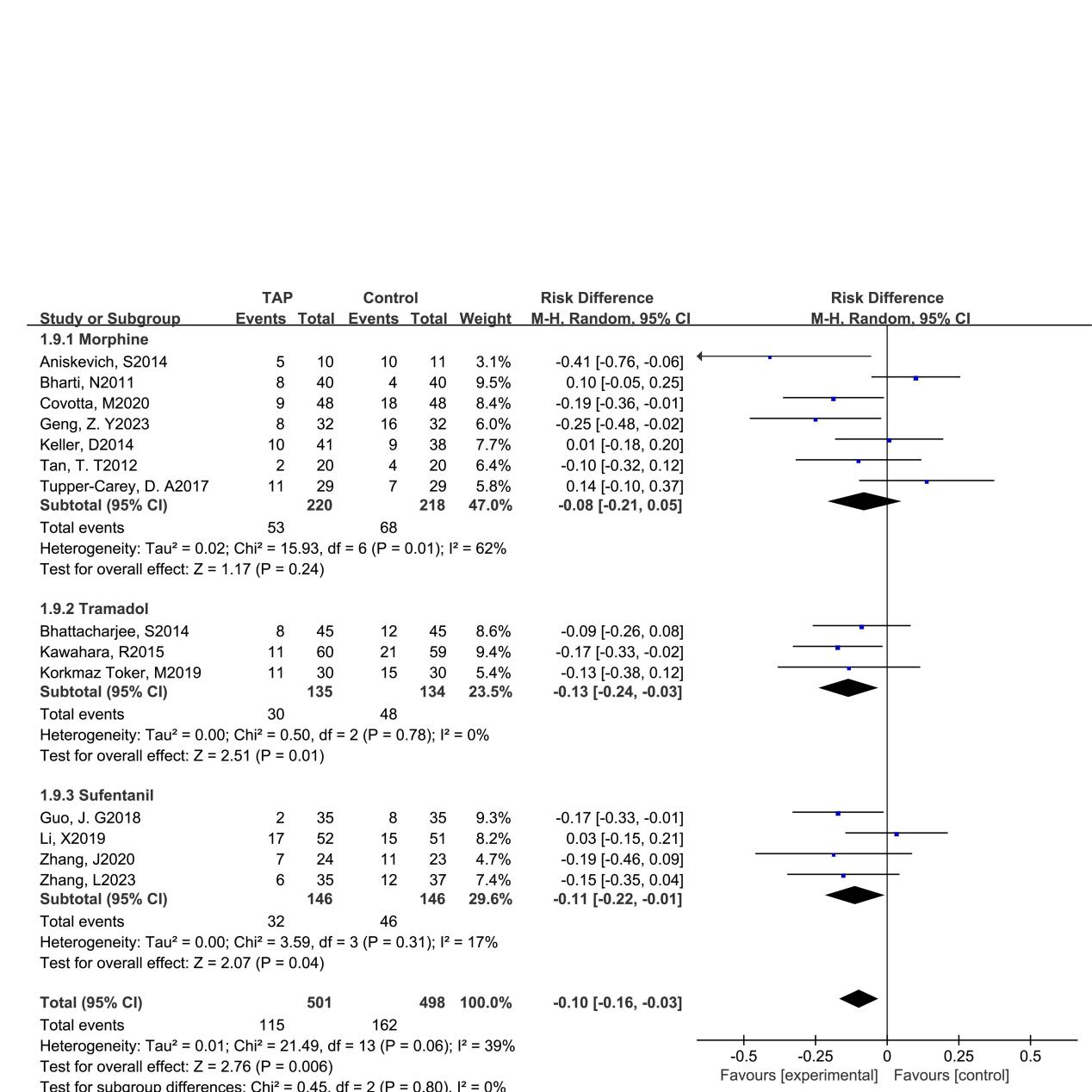


**Supplementary figure 5.** Intraoperative cases with antiemetic (A) or satisfaction degree(B).


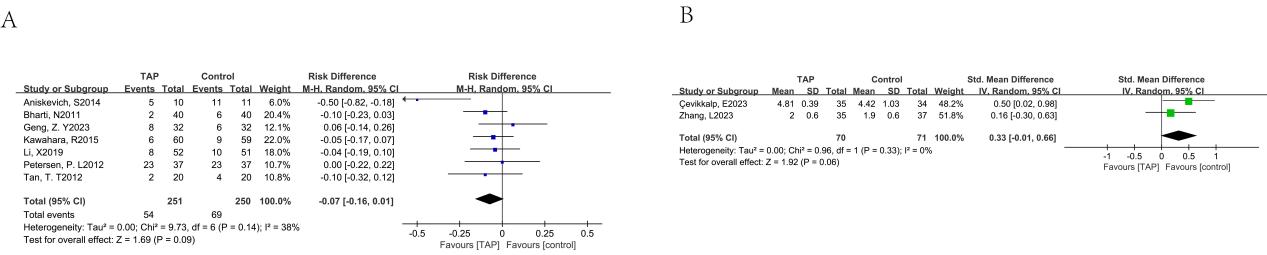


**Supplementary table 1.** GRADE summary of efficacy of transversus abdominis plane block on postoperative nausea and vomiting.

| **Quality assessment** | | | | | | | | | | | **No of patients** | | | | **Effect** | | | **Certainty** | | **Importance** |
| --- | --- | --- | --- | --- | --- | --- | --- | --- | --- | --- | --- | --- | --- | --- | --- | --- | --- | --- | --- | --- |
|  |  |  |  |  |  |  |  |  |  |  |  |  |  |  |  |  |  |  |  |  |
| **No of studies** | **Design** | **Risk of bias** | | **Inconsistency** | **Indirectness** | | | **Imprecision** | | **Other considerations** | **TAP** | | **Control** | | **Relative**  **(95% CI)** | | **Absolute** |  |  |  |
| **Nausea** | | | | | | | | | | | | | | | | | | | | |
| 23 | Randomized trials | serious^1^ | | serious^2^ | no serious indirectness | | | no serious imprecision | | reporting bias^3^ | 181/893  (20.3%) | | 266/883  (30.1%) | | RD -0.10  (-0.15 to -0.05) | | 99 fewer per 1000 (from 51 fewer to 151 fewer) | ⨁OOO  VERY LOW | | CRITICAL |
|  |  |  |  |  |  |  |  |  |  |  |  |  |  |  |  |  | 97 fewer per 1000 (from 50 fewer to 147 fewer) |  |  |  |
| Vomiting | | | | | | | | | | | | | | | | | | | | |
| 11 | Randomized trials | | Serious^4^ | serious^2^ | | no serious indirectness | no serious imprecision | | reporting bias^3^ | | | 50/351  (14.2%) | 59/358  (16.5%) | RD -0.01  (-0.05 to 0.03) | | 8 fewer per 1000 (from 49 fewer to 30 more) | | | ⨁OOO  VERY LOW | CRITICAL |
|  |  |  |  |  |  |  |  |  |  |  |  |  |  |  |  | 7 fewer per 1000 (from 41 fewer to 24 more) | | |  |  |

#### Explanations

1. Lack of blinding in Hutchins, J 2014 and Reisener, M.J 2021 study; Lack of allocation concealment in Aniskevich, S 2014, Cevikkalp, E 2023, Covotta, M 2020, Guo, J. G 2018, Hutchins, J 2014, Kawahara, R 2015, Keller, D 2014, Kim, M. G 2014, Li, X 2019, Lochel, J 2021, Lu, X 2020, Ma, J 2018, McKeen, D. M 2014, Petersen, P. L 2012, Reisener, M. J 2021, Sivapurapu, V 2021, Skjelsager, A 2013, Soltani Mohammadi, S 2014, Tupper-Carey, D. A 2017, Zhang, L 2023 study

2. CIs showed minimal or no overlap

3. The publication bias was not assessed because of the limit of the amount of included studies, and which strongly suspected all plausible residual confounding would reduce the demonstrated effect

4. Lack of allocation concealment in Aniskevich, S 2014, Guo, J. G 2018, Lochel, J 2021, Lu, X 2020, McKeen, D. M 2014, Petersen, P. L 2012, Skjelsager, A 2013, Zhang, L 2023 study

**Supplementary table 2.** Description of opioid usage and dose, rescue antiemetics and nausea scores.

| **Author** | **Year** | **Comparisons** | **Sex**  **(M/F)** | **Smoker** | **The usage of opioids** | **PCA** | **Dose of opioids** | **Rescue antiemetics** | **Nausea scores or cases** |
| --- | --- | --- | --- | --- | --- | --- | --- | --- | --- |
| Aniskevich, S | 2014 | TAP 0.5% ropivacaine 100mg | 6/4 |  | Anesthesia was maintained with fentanyl (up to 250 µg), fentanyl boluses to a maximum of 250 µg. Postoperatively, participants were given intravenous patient-controlled morphine analgesia dosed at 1 mg with an 8-minute lockout. | morphine |  |  |  |
|  |  | Control saline 20ml | 6/5 |  |  |  |  |  |  |
| Bharti, N | 2011 | TAP 0.25% bupivacaine 50mg | 14/6 |  | Anesthesia was induced with IV morphine sulfate 0.15 mg/kg | morphine | Morphine: 6.45 ± 3.26 mg |  |  |
|  |  | Control saline 20ml | 14/6 |  |  |  | Morphine: 17.55 ±5.78mg |  |  |
| Bhattacharjee, S | 2014 | TAP bupivacaine 0.25% 0.5 ml/kg | 0/45 |  | Ten min after TAP block, all patients received a standardized general anesthesia with fentanyl 1 mcg/kg, patients were shifted to a postoperative analgesic regimen of injection tramadol IV 2 mg/kg 8 hourly and injection paracetamol 6 hourly up to 24 h. | tramadol | Intraoperative fentanyl  Requirement:  81 (46-122) mg | Time to requirement of  first analgesic in minute 290 (60-1440) |  |
|  |  | Control l saline 0.5 ml/kg | 0/45 |  |  |  | Intraoperative fentanyl  requirement :  114 (74-183) mg | 16 (0-70) |  |
| Çevikkalp, E | 2023 | TAP 0.25%bupivacaine | 11/24 | 9 | For the induction of anesthesia, 0.2 mg/kg fentanyl Acetaminophen 1 mg was gradually administered every 6 hours depending on the level of pain, while diclofenac sodium was intravenously administered every 12 hours when required. Intravenous tramadol 50 mg was given as a rescue analgesic to patients with a pain score higher than 4 |  |  | Rescue analgesic 3 cases |  |
|  |  | Control:saline 0.5 ml/kg | 7/27 | 10 |  |  |  |  |  |
| Covotta, M | 2020 | TAP 0.5%ropivacaine 150mg | 30/18 |  | Anesthesia was induced with propofol 2 mg/kg and fentanyl 1 mcg/kg. If needed, additional fentanyl was provided to control blood pressure.. The dose of morphine was set at 0.02 mg/kg with a time-lock interval of 15 minutes. （PCA) | morphine | Intraoperative fentany: 288 ± 68 μg; Morphine consumption up to 24 h :10.6 ±4.6 mg |  |  |
|  |  | Control nothing | 22/26 |  |  |  | Intraoperative fentanyl: 296 ± 50μg; Morphine consumption up to 24 h :14.1 ± 4.4 mg |  |  |
| Geng, Z. Y | 2023 | TAP 0.375%ropivacaine 75mg | 0/32 |  | All patients received furbiprofen 50mg IV immediately before skin incision as preemptive analgesia.IV boluses of morphine 1–2 were given as a rescue opioid for NRS pain score≥4. After discharge from the PACU, a morphine intravenous patient-controlled analgesia (PCA) was provided as rescue analgesia (no background infusion, 1mg bolus with a 6-min lockout interval). Another furbiprofen 50mg IV was administered 6h after surgery. All patients received regular oral acetaminophen (650mg every 8h), and oral celecoxib (200mg every 12h) on postoperative day (POD) 1 and 2. | morphine | Intraoperative remifentanil : 6.1±1.3 μg·kg^−1^·h^−1^;  Intraoperative sufentanil : 0.3±0.1 μg/kg;  Morphine consumption at 0–24h: 5 (2-9) mg  Morphine consumption at 24–48h: 1 (0-1) mg | Rescue antiemetics 0–48h (n/%) 8 cases (25.0%) | 3.4±0.6 |
|  |  | Control saline 20ml | 0/32 |  |  |  | Intraoperative remifentanil: 5.9±0.8 μg·kg^−1^·h^−1^; Intraoperative sufentanil : 0.3±0.1 μg/kg; Morphine consumption at 0-24h: 8.5 (5-12.8) mg; Morphine consumption at 24-48h : 0 (0–2) mg | Rescue antiemetics 0–48h (n/%) 6 cases (18.8%) | 3.5±0.5 |
| Guo, J. G | 2018 | TAP 0.375%ropivacaine 75mg | 25/10 |  | Sufentanil (0.2-0.6 μg/kg) If MAP or HR of the patient increased up to 20% of the initial value, intravenous sufentanil 0.05 μg/kg was administered. Repeated doses of sufentanil were given  every 5 min to keep the blood pressure around the pa tient’s baseline values.PCA was set to deliver a bolus of 2 μg of sufentanil with a lockouttime of 10 min with a continuous infusion at 1 μg/h. | sufentanil |  |  |  |
|  |  | Control saline 20ml | 23/12 |  |  |  |  |  |  |
| Hutchins, J | 2014 | TAP 0.25%Bupivacaine 37.5mg | 0/30 |  | NOT mention |  |  |  |  |
|  |  | Control nothing | 0/30 |  |  |  |  |  |  |
| Kawahara, R | 2015 | TAP 0.375%ropivacaine 75mg | 0/60 |  | 0.1 μg/kg/min of remifentanil for analgesia postoperatively, patients received a PCA setting of a bolus of 12 mg tramadol with 10 min lockout time without basal infusion. The solution was prepared as 300 mg (6 mL) of tramadol diluted with 44 mL of isotonic saline. | tramadol | Remifentanil: 1.7 (0.7-4.0) mg  Tramadol (PCIA) (0-6H): 18 (0-144) mg |  |  |
|  |  | Control saline 20ml | 0/59 |  |  |  | Remifentanil: 2.1 (0.8-8.5) mg; Tramadol (PCIA) (0-6H) :24 (0-84) mg |  |  |
| Keller, D | 2014 | TAP 0.5%bupivacaine 150mg | 18/23 |  | In the postoperative holding area, a morphine patient-controlled analgesia (PCA) was initiated for all patients. All opioids in the postanesthesia care unit (PACU) were self-administered via the PCA. On the nursing floor, patients receive gabapentin 300mg orally twice a day for 72 hours (or while in the hospital), Toradol 15mg intravenously every 6 hours for 48 hours, and Tylenol 1 g every 6 hours. On postoperative day 1, the morphine PCA is discontinued, Tylenol is transitioned from intravenous to oral, and pain man agement is initiated with Oxycodone 10mg orally every 6 hours as needed. | morphine | Intraoperative fentanyl: 163.18±76.65μg; Total opioids  in PACU 0.80±0.52 mg |  |  |
|  |  | Control saline 30ml | 16/22 |  |  |  | Mean intraoperative fentanyl: 146.43±61.28 μg; Total opioids in PACU :1.79±0.72 mg |  |  |
| Kim, M. G | 2014 | TAP 0.375% ropivacaine112.5mg | 31/2 |  | Anesthesia was induced remifentanil 1 μg/kg.BIS at 40-60 by adjusting the infusion rate of remifentanil.Toward the end of the surgery, we stopped the infusion of remifentanil and recorded the amount used, and then administered propacetamol hydrochlo ride 1 g mixed with normal saline.NRS>5 we administered fentanyl 50 μg intravenously. If re quired by the patient or the NRS was greater than 5, fentanyl 50μg was likewise administered after an interval of 10 min. |  | Remifentanil requirement: 0.05 ± 0.03µg/kg/min  Fentanyl requirement :39.4 ± 46.4µg Ketorolac: 3.6 ± 12.5mg |  |  |
|  |  | Control nothing | 33/4 |  |  |  | Remifentanil requirement : 0.06 ± 0.03µg/kg/min;  Fentanyl requirement: 74.3 ±49.5µg;Fentanyl requirement: 12.2 ± 19.3µg |  |  |
| Korkmaz Toker, M | 2019 | TAP 0.375%bupivacaine 150mg | 0/30 |  | Remifentanil (intravenous [IV], 0.5-1mg/kg per min)Twenty minutes before the completion of surgery, all patientsreceived dexketoprofen trometamol (75mg), ondanse ron (0.1mg/kg), and a loading dose of tramadol (1mg/kg). Following loading dose of tramadol, the tramadol PCA was administered. All 2 groups received the same tramadol PCA. IV tramadol patient controlled analgesia (PCA) (basal infusion: 10mg/h, bolus 20mg every 15 minutes, maximum dose 90mg/h) In the case of a VAS score of 4 or more, standard postoperative analgesia regimen consisted of intramuscular dexketoprofen trometamol (75mg), 12-hourly,was carried out. | tramadol | Remifentanil consumption: 230 (217.5–240)mg  Tramadol consumption 24th hour: 333.3±34.9mg |  | 2 (1.00-2.25) |
|  |  | Control saline 40ml | 0/30 |  |  |  | Remifentanil consumption: 230 (212-250)mg  Tramadol consumption 24th hour: 355.3±28.1mg |  | 2 (2-3) |
| Li, X | 2019 | TAP 0.4%ropivacaine 120mg | 32/20 |  | Anaesthesia was induced with sufentanil,Anaesthesia was maintained with continuous infusion of remifentanil (and intermittent sufentanil) or sufentanil,At 30 min before the end of sur gery, 50 mg of flurbiprofen axetil and 5 mg of tropisetron were administered intravenously.PCA was established with 1.25 μg/ml sufentanil and programmed to administer a background rate of 0.5 ml/h and an on demand bolus of 4 ml every 10 min, together with a rigorous rescue analgesia plan | sufentanil | Opioid consumption during surgery Sufentanil : 23 (20- 30)μg; Remifentanil: 607 (428-818)μg ; Morphine equivalent dose: 40.3 (20.9- 59.0)mg ;Opioid consumption within 24 h after surgery Sufentanil: 40 (24-80) μg; Morphine equivalent dose: 13.2 (8.0- 26.6)mg | 19 cases (36.5%) |  |
|  |  | Control saline 30ml | 31/20 |  |  |  | Opioid consumption during surgery Sufentanil: 20 (15-38)μg ; Remifentanil, 600 (502-794)μg ; Morphine equivalent dose: 35.0 (18.0-49.6)mg; Opioid consumption within 24 h after surgery Sufentanil: 33 (23- 65) μg ; Morphine equivalent dose: 10.8 (7.8-21.7)mg | 12 cases (23.5%) |  |
| Lochel, J | 2021 | TAP 0.75%ropivacaine 150mg | / |  | Anaesthesia was induced fentanyl (0.5-1 µg/kg) The anaesthesia was maintained fentanyl administered for intraoperative analgesia. |  |  |  |  |
|  |  | Control nothing |  |  |  |  |  |  |  |
| Lu, X | 2020 | TAP 0.25%ropivacaine 100mg | 59/4 |  | For induction, sufentanil (0.5 μg/kg) For maintenance, remifentanil (0.05-0.2 μg·kg-1·min-1) PCA:One hundred milliliters of 1 mg/mL oxycodone was used in the analgesic pump. The flow rate of the pump was 1 mL/h with a bolus of 2 mL and 5 min of lockout time. | oxycodone | Remifentanil dose :736.5±417.8 μg |  |  |
|  |  | Control nothing | 45/18 |  |  |  | Remifentanil : dose738.2±347.6 μg |  |  |
| Ma, J | 2018 | TAP 0.375% ropivacaine 75mg | 18/11 |  | Anesthesia was induced using 0.4 µg/kg sufentanil, general anesthesia was maintained by propofol and remifentanil |  | Remifentanil infusion : 0.16±0.05ug kg^−1^min^−1^ | Time to first rescue analgesia 324±72 min |  |
|  |  | Control saline 20ml | 17/11 |  |  |  | Remifentanil infusion: 0.21±0.05ug kg^−1^ min^−1^ | Time to first rescue analgesia 126±34 min |  |
| McKeen, D. M | 2014 | TAP 0.25%ropivacaine 100mg | 0/35 |  | The spinal anesthetic fentanyl 15 μg, oxycodone 2.5-5 mg q6 h prn. converted to oral morphine equivalents at a ratio of 1:1.5 oxycodone to morphine |  | Morphine Equivalents: 15.5± 20.2mg |  |  |
|  |  | Control saline 40ml | 0/39 |  |  |  | Morphine Equivalents 13.4± 14.6mg |  |  |
| Petersen, P. L | 2012 | TAP 0.5%ropivacaine 100mg | 9/28 |  | Anesthesia was induced with remifentanil 0.4 mL/kg/h (0.6 mg/mL) Anesthesia was maintained with remifentanil (fixed rate 0.4 mL/kg/h). Ten minutes before the end of surgery, all patients received IV sufentanil 0.2 μg/kg. The initial morphine dose was 5 mg and the following doses were 2.5 mg with a minimum of 10 minutes between doses. | morphine | Remifentanil : 2.8 ±1.0mg Sufentanil : 15.3 ±2.8μg |  | 0 (0–2) |
|  |  | Control saline 20ml | 12/25 |  |  |  | Remifentanil : 2.4 ±0.7mg Sufentanil : 15.7±3.5μg |  | 0 (0–3) |
| Reisener, M. J | 2021 | TAP 0.5% bupivacaine 100-150mg | 54/75 | 10 | Opioids on an as-needed basis (tramadol 50 mg every 4 h, or oxycodone 5–10 mg every 4 h, according to pain scores) |  | Opioid consumption PACU to 24 h  post-surgery (MED): 33.1 (15.0- 67.5)mg |  |  |
|  |  | Control nothing | 61/60 | 9 |  |  | Opioid consumption PACU to 24 h  post-surgery (MED): 37.5 (15.0-78.7)mg |  |  |
| Sivapurapu, V | 2021 | TAP 0.25%levobupivacaine 45mg | 28/2 |  | Fentanyl 2 mcg kg^−1^ i.v. Increments of fentanyl 0.5 mcg kg^−1^ i.v. were supplemented if further analgesia was required intraoperatively by monitoring for a 20% increase in heart rate or mean arterial pressure.administered at induction. tramadol 50 mg i.v. was administered if NRS >4. |  | Total analgesia :1.5 ±1.2mg kg^−1^ ; Fentanyl (total ) 120±40mcg |  |  |
|  |  | Control nothing | 26/4 |  |  |  | Total analgesia : 1.85 ±0.9 mg kg^−1^; Fentanyl (total ) 130±33mcg |  |  |
| Skjelsager, A | 2013 | TAP 0.75% ropivacaine | 23/0 |  | Surgery was performed in general anaesthesia using remifentanil (0.03 mg/kg/h).Thirty minutes before the end of surgery, all patients received morphine 0.15 mg/kg intravenously.PCA:morphine. The pump was set to a bolus of 2.5 mg and 10-min lockout, with no background infusion. If this was inadequate, additional boluses of 2.5 mg IV morphine was given by a nurse the first post-operative hour until adequate analgesia was obtained. | morphine | Remifentanil (median and IQR) : 5.5 (4.4-7.4)mg Morphine (median and IQR): 12.5 (11.4-14)mg |  |  |
|  |  | Control | 24/0 |  |  |  | Remifentanil (median and IQR) : 5.9 (5.2-6.9) mg ; Morphine (median and IQR): 12.8 (11.6-13.7) mg |  |  |
| Soltani Mohammadi, S | 2014 | TAP 0.25%Bupivacaine 37.5mg | 12/10 |  | Anesthesia was induced with IV thiopental sodium 5 mg/kg and fentanyl 3 μg/kg. IV fentanyl 1 μg/kg as required.To deliver 1 mg of morphine on demand every10 minutes until NRS = 3.0. There was no continuous infusion. |  | The 24-hour morphine consumption :10.8 ± 9.5mg;  Intraoperative fentanyl consumption: 120 ± 20 μg |  |  |
|  |  | Control saline 15ml | 14/8 |  |  |  | The 24-hour morphine consumption: 41.2 ± 3.8mg; Intraoperative fentanyl consumption :358 ± 24 μg |  |  |
| Tan, T | 2012 | TAP 0.25%levobupivacaine 50mg | 0/20 |  | Analgesia was provided with intravenous morphine 0.15 mg/kg. PCA system using intravenous morphine(bolus dose 1 mg, lockout interval 5 min, maximum dose 40 mg in 4 h). | morphine | Intraoperative morphine: 10.7±0.4mg |  |  |
|  |  | Control nothing | 0/20 |  |  |  | Intraoperative morphine: 10.6±0.4mg |  |  |
| Tupper-Carey, D. A | 2017 | TAP 0.5%bupivacaine 50mg | 21/8 |  | An intravenous morphine PCA system, which was programmed to a standard regime (1-mg bolus, five-minute lockout, maximum hourly dose of 5 mg and no background infusion), was administered to each patient. .Morphine PCA was continued for a minimum of 12 hours postoperatively for all patients; Morphine PCA was continued if pain scores were > 4 or if the pain team decided that continuation was necessary on clinical grounds. | morphine | PCA morphine consumption in first 12 hr : 9.79 ± 8.09mg; Total equianalgesic morphine consumption in 24 hr :11.31 ± 8.66mg; Total dose of intraoperative fentanyl: 128.71 ± 36.79mcg |  |  |
|  |  | Control saline 10ml | 25/4 |  |  |  | PCA morphine consumption in first 12 hr : 11.45 ± 7.64mg;  Total equianalgesic morphine consumption in 24 hr: 13.38 ± 8.72mg;  Total dose of intraoperative fentanyl : 160.89 ± 47.3mcg |  |  |
| Zhang, J | 2020 | TAP 0.3%ropivacaine 180mg | 12/12 |  | Anesthesia was induced with intravenous sufentanil (0.5 µg/kg to a maximum of 50 µg),general anesthesia was maintained through d remifentanil (0.1–0.4 µg/kg/min). 0.2 µg/kg sufentanil was added before the end of the operation.The PCIA pump was programmed in an only-bolus mode without a basal rate, allowing for a 2 µg sufentanil bolus injection every 5 min with a maximum of 40 µg every 4h. Sufentanil was administered on demand if the VAS scoreswere >3 on assessment; | Sufentanil | Sufentanil consumption 48H: 33 (10-54)μg |  | 0: 17 (70.83%)  1: 4 (16.67%)  2: 1 (4.17%)  3: 2 (8.33%) |
|  |  | Control saline 60ml | 13/19 |  |  |  | Sufentanil consumption : 48H78 (52-94)μg |  | 0: 12(52.17%)  1: 5 (21.74%)  2: 2 (8.69%)  3: 4 (17.40%) |
| Zhang, L | 2023 | TAP 0.375%ropivacaine 150mg | 0/35 |  | Each patient was administered analgesics using a PCA pump containing sufentanil (200 μg) diluted by saline to a total volume of 200 mL after discharge from the PACU for 72 h. The device was set to deliver a basal infusion of 2 mL h^−1^during the first 3 h, and bolus doses of 0.5 mL with a 15 min lockout period was given as needed for 72 h. Intravenous sufentanil (5-10 μg) as rescue analgesia was available as needed for breakthrough pain. | sufentanil | Method of sufentanil application By PCA :  0-6h :8.8±2.5μg  6-12h: 6.4±2.8μg  12-24h: 10.9 ±4.0μg  24-48h :11.5 ±4.4μg  48-72h :7.7±3.2μg  By titration in the PACU:  2.1 ±2.1μg |  |  |
|  |  | Control saline 40ml | 0/37 |  |  |  | Method of sufentanil application By PCA :  0-6h: 12.3±2.6μg  6-12h : 8.0±2.8μg  12-24h: 12.6 ±4.5μg  24-48h: 12.8±5.0μg  48-72h: 8.0 ±2.9μg  By titration in the PACU:  4.1 ±2.5μg |  |  |

**Notice: Previous PONV/ Motion Sickness not mentioned**
